# Challenges and Controversies in Human Mesenchymal Stem Cell Therapy

**DOI:** 10.1155/2019/9628536

**Published:** 2019-04-09

**Authors:** Barbara Lukomska, Luiza Stanaszek, Ewa Zuba-Surma, Pawel Legosz, Sylwia Sarzynska, Katarzyna Drela

**Affiliations:** ^1^NeuroRepair Department, Mossakowski Medical Research Centre, Polish Academy of Sciences, Warsaw, Poland; ^2^Department of Cell Biology, Faculty of Biochemistry, Biophysics and Biotechnology, Jagiellonian University, Krakow, Poland; ^3^Department of Orthopedics and Traumatology, 1st Faculty of Medicine, Medical University of Warsaw, Warsaw, Poland

## Abstract

Stem cell therapy is being intensely investigated within the last years. Expectations are high regarding mesenchymal stem cell (MSC) treatment in translational medicine. However, many aspects concerning MSC therapy should be profoundly defined. Due to a variety of approaches that are investigated, potential effects of stem cell therapy are not transparent. On the other hand, most results of MSC administration *in vivo* have confirmed their safety and showed promising beneficial outcomes. However, the therapeutic effects of MSC-based treatment are still not spectacular and there is a potential risk related to MSC applications into specific cell niche that should be considered in long-term observations and follow-up outcomes. In this review, we intend to address some problems and critically discuss the complex nature of MSCs in the context of their effective and safe applications in regenerative medicine in different diseases including graft versus host disease (GvHD) and cardiac, neurological, and orthopedic disorders.

## 1. Introduction

Mesenchymal stem cells (MSC) are of clinical interest because of their potential use in autologous transplantation. A lot of clinical trials using MSCs have been accomplished, and many others are being under examination. Recent reports demonstrated that more than two thousand patients received autologous or culture-expanded allogeneic MSCs for the treatment of different diseases [1]. In most cases, MSC therapy was quite efficient. However, the potential risk of MSC transplantation should be considered in terms of the long-lasting observations. Numerous reports from *in vitro* and *in vivo* studies provided the evidence about MSC differentiation into certain cell types [2]. However, a growing evidence from recent studies strongly suggests to focus on MSC paracrine properties including the release of extracellular vesicles containing numerous mRNAs, regulatory miRNAs, multiple bioactive proteins and compounds [3], and the production and secretion of a large number of regulatory substances rather than MSC direct differentiation and cell replacement [4]. The main therapeutic effects of MSCs are now attributed to the stimulation of several endogenous repair processes in injured tissues *in vivo* by secreted factors as well as the modulation of immune response, which translates into a positive outcome of MSC-based therapies.

Another important aspect is the cellular heterogeneity of MSCs, which makes consistent conclusions about MSC therapeutic potential difficult, because the obtained results are frequently variable and may depend on the different MSC origin as well as harvesting and culture procedures [5]. At the same time, it makes MSCs a very interesting type of cells to be studied due to their complex nature. So far, there is no precise MSC definition, and already existing definitions only partially reflect the functional properties of these cells [6]. Due to the great interest in MSCs, a large number of publications explore the biological properties of these cells [7]. Several *in vitro* studies are aimed at defining compound and overlapping molecular mechanisms that may be involved in therapeutic MSC action *in vivo*. Besides these facts, the results of other multiple *in vitro* studies pave the way to possible modifications of the ex vivo culture environment and MSCs themselves to further increase their regenerative potential [7], and consequently to achieve better results in *in vivo* studies.

**Figure 1:**
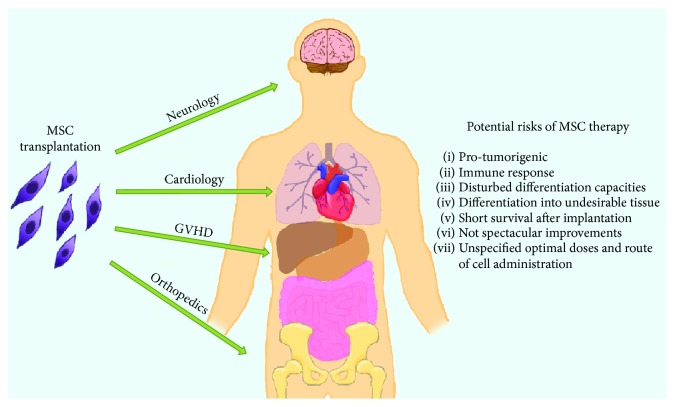
The scheme of potential risks of adverse events during MSC transplantation.

In the present article, we discuss the potential side effects of exogenous MSC administration *in vivo*. In particular, we focus on GvHD [8] and cardiologic [9], neural [10], and orthopedic [11] patients, where MSC administration may have limited beneficial effects and provide some side effects for the recipient (Figure 1). We believe that, discussing some adverse aspects of MSC activity after their transplantation, we create a more reliable and complete view of MSC role in regenerative medicine.

## 2. Heterogeneity of MSCs

There are reports that demonstrated that the quality of the human MSC product depends on the isolation and culture methods as well as the age, genetic traits, and medical history of the donor [12–15]. It seems that the most important factor to consider is the age of a donor so the autologous transplantation can have some limitations. The challenging issue is how to expand MSCs from elderly patients to obtain an efficient number of therapeutic cells. Moreover, it is difficult to isolate an effective population of MSCs from patients with some diseases including diabetes, rheumatoid arthritis, and other inflammatory diseases, thus regarding autologous reasons (where the cells isolated from the patient might be affected by the disease); the researchers suggest possible loss of therapeutic function by these cells [15]. Baptista and colleagues demonstrated that MSCs obtained from people with obesity have significantly impaired proliferation and differentiation potential [16]. However, due to some problems with autologous sources, we should consider immunological aspects of allogeneic MSC transplantation. Historically, MSCs have been shown to exhibit low immunogenic potential *in vitro* because of their limited expression of MHC I molecules, the lack of MHC II expression, and costimulatory molecules. Recent studies suggest that MSCs may not be “immune privileged” as assumed previously. It was shown that MSCs are no longer considered immunologically silent *in vivo* [17, 18]. Moreover, use of allogeneic MSCs has some limitations, considering risk factors including immunological response [19].

## 3. Potential Side Effects of Exogenous MSCs after Their Administration *In Vivo*

The past decade has seen an explosion of research which directed at evaluation of mesenchymal stem cells as a cellular therapy in different debilitating diseases; however, a comprehensive analysis is needed to explain the discrepancies in clinical trials examining MSC treatment. Many negative effects are probably unreported, but they can occur. Stem cell therapies are complicated and can have serious side effects. A case report was published recently demonstrating that the patient developed a large tumor-like mass inside the spinal cord after 8 years of olfactory mucosa cell transplantation [20]. Although the trial was performed with the use of different adult stem cells than the MSC type, one should take into account that the potential mechanism of neoplastic transformation could be similar in case of MSC transplantation. Thus, more control investigations have to be performed with MSC samples prior to their transplantation to the specific niches in different tissues. In the following subsections of review, we will focus on side effects of MSCs therapy in different diseases.

### 3.1. MSCs as Advanced Therapy Medical Products

MSCs are now determined as Advanced Therapy Medicinal Products (ATMP) and guidelines from the American Code of Federal Regulation of the Food and Drug Administration and the European Medicines Agency, and the network of national agencies defines the requirements for suitable production of these cells. These guidelines, generally called “Good Manufacturing Practices” (GMP), consist of many recommendations about cell culture procedures including validation and quality control to ensure optimal reproducibility, efficiency, and safety of the final medical product. Another classification, defined by the Food and Drug Administration guidelines, defines cultured cells as “minimally” and “more than minimally” manipulated which describe procedures “that might alter the biological features of the cells.” However, minimal manipulation conditions have not been precisely defined for the collection and isolation of various sources of MSCs [21–23]. Most of cell culture protocols that have been reported so far are inadequate, because of the use of supplemented cell culture media, enzymatic treatment, and long-term cell expansion that are known to change the quality of MSCs [21]. The results published by Codinach and collaborators showed bioprocess engineering application for bone marrow-derived MSC (BM-MSC) isolation, expansion, validation, and production for clinical use. The authors tested 48 batches of BM samples from the iliac crest for autologous transplantations [20]. The manufacturing process contained diverse steps including collection, isolation, trypsinisation, and all quality control that is needed for confirmation of functionality, safety, and potency of MSCs. In conclusion, Codinach et al. confirmed that all steps are effective and reproducible and most importantly safe for clinical use [23]. Nevertheless, validation of optimal isolating and culturing protocols according to GMP requirements is still needed.

### 3.2. MSCs to Treat or Prevent Graft-versus-Host Disease

Graft-versus-host disease (GvHD) accompanies allogeneic hematopoietic stem cell transplantation (HSCT) in many patients. In GvHD treatment, the corticosteroids are used; however, this therapy is not effective in all patients [8]. Immunomodulatory properties of MSCs reported in experimental studies suggest their application in GvHD treatment. Indeed, recently MSC transplantation was performed to prevent or to treat GvHD especially in those patients who are unresponsive to steroids [24]. However, despite of MSC applications in many clinical trials, the controversy still exists for the profits of such a therapy. Although the reduction of inflammatory processes is noticed after MSC implantation, downregulation of immune response could augment the chance of infections, especially in patients receiving immunosuppressive therapy after HSCT [25]. It was reported that the infusion of MSCs might dangerously constrain antimicrobial immune response [26]. von Bahr et al. have shown enhanced complications in a retrospective study in GvHD patients due to the infections in a short period of time after different source derived (BM/PBSCs/CB) MSC transplantation, causing the mortality to reach 54% [27]. The infection-related mortality was high even after GvHD determination. It may be related to the long immunosuppressive effect of MSCs. The clinical trial announced by Ning et al. showed that the occurrence of acute and chronic GvHD in patients after MSC transplantation was lower than in patients without MSC graft, but the episodes of severe infections were higher in patients who have received HSCT and bone marrow-derived MSCs than in the control group not receiving MSCs. Among patients, two of them manifested CMV interstitial pneumonia and/or fungal infection [28]. Forslöw et al. suggest an increased susceptibility to pneumonia observed in patients with GvHD after MSC infusion [29]. The retrospective study of patients with steroid-refractory GvHD receiving MSCs announced high CMV peak viral load [27]. This is the contradiction with earlier *in vitro* experiments which depicted that cytotoxic T cells against CMV were restricted to BM-MSC effect [30]. Recently, Thanunchai et al. have postulated that in viral infections human BM-MSCs might also act as viral transmitters [31]. Moreover, in different experimental models it was shown that BM-MSCs encourage tumor growth by modulating the tumor microenvironment [32, 33]. In a pilot clinical study using MSCs to prevent GvHD in patients with hematologic malignancies, MSCs decreased GvHD development but the relapse rate in patients was over the control group. Out of 10 patients, 6 of them in the MSC group suffered from tumor relapse in comparison to 3 of 15 in the control group not receiving MSCs [28]. The protumorigenic effects revealed by MSCs are probably related to their immunosuppressive properties, the modulation of tumor stroma, and their ability to transform themselves into malignant cells. However, the experiments confirming the tumorigenic potential of MSCs were conducted on rodent models. Up to now, there is no existing data displaying malignant transformation of human MSCs. Moreover, it is not clear whether human MSC aneuploidy is not related with senescence or transformed population of cells [34]. The existent data concerning the direct in vitro transformation of MSCs were retracted due to contamination with other cell lines. It has been also reported that transplantation of MSCs from diverse sources (BM, placenta, or umbilical cord blood) to haploidentical mice did not prevent or treat GvHD [35]. The suggestion exists that MSCs may lose their immunosuppressive properties in mismatched settings, which was proved on murine cells [36]. Furthermore, the Muroi et al. study showed that grafted BM-MSCs in the phase II/III study for acute GvHD does not protect the development of chronic GvHD [37].

Based on the above mentioned studies, it needs to be highlighted that MSC transplantation for GvHD prevention or therapy is relatively safe and efficient in steroid-refractory GvHD, but infections remain a major risk of patients. Furthermore, it was shown that MSCs transplanted for established GvHD can cause an increased relapse [28]. In the recent paper published by Ringden et al.'s group, the authors mention several adverse effects occurring after transplantation of decidual placenta-derived MSCs in the treatment of GvHD. Among them, relapse; pneumonia; bacterial, viral, and fungal infection; and graft failure are listed [38]. It seems that the new strategy supporting a high rate effect of MSCs against GvHD with a low adverse effect to the patient is warranted in large-scale randomized studies. The research carried by different laboratories focuses on the development of new MSC drugs. One of the first registered MSC-based drugs recommended to use for treatment of GvHD was Prochymal. Recently, Mesoblast recruited patients for clinical phase 3 for treatment of GvHD and others like chronic heart failure and chronic low back pain. The company developed the strategy of isolation and banking of BM-MSCs derived from healthy donors. However, this product is not yet approved by FDA. Similarly iPSC-derived MSCs were proposed by Cynata's therapeutics. Investigation is within clinical phase 1; however, it is still hard to evaluate the results due to the early stage of research.

### 3.3. MSCs in Cardiology

Cardiovascular diseases (CVDs) affecting both heart tissue and circulatory system, especially blood vessels, represent today one of the main causes of mortality in Western countries. According to the report from the American College of Cardiology, CVDs caused close to 17.3 million deaths worldwide in 2008 and the predictions indicate an increase in the mortality to about 25 million deaths per year by 2030 [39]. Since heart tissue exhibits limited endogenous potential for cardiac cell proliferation and repair, multiple stem cell-based therapeutic approaches have been already tested in several preclinical animal models and clinical trials in humans, indicating potential beneficial effects of such treatment on the heart anatomy and functions in different CVDs [40, 41]. Experimental cardiology represents today the clinical field greatly relaying on various novel approaches employing cell therapy products that may enhance cardiac tissue repair and regeneration [42].

Adult stem cells including MSCs have been indicated as one of the most promising therapeutic cells for myocardial repair and have been also widely used as potential therapeutic cells in several cardiovascular diseases [43, 44]. Although MSCs exhibit great prochondrogenic, osteogenic, and adipogenic differentiation potential, several studies provided evidence that in optimal culture conditions *in vitro* or in cardiac *in vivo* niche, MSCs may also give rise to other highly specialized tissue types including cardiomyocytes and endothelial cells [45–47]. Considering these findings, MSCs have been extensively tried as a source of cells replacing damaged myocardial tissue *in vivo*, in both acute and chronic heart injury events, confirming the trans-differentiation capacity of MSCs into cardiac and endothelial cells [43, 44]. Nevertheless, the differentiating capacities of MSCs into functional endothelial and cardiac cells *in vivo* have not been well fully confirmed. Moreover, it is hard to confirm the presence of transplanted, integrated cells in any tissue in vivo, due to the lack of specific MSC markers [48]. However, growing recent evidence from multiple laboratories strongly indicates predominant paracrine activity of MSCs after transplantation, promoting cardiac cell survival, proliferation, and differentiation by MSC-derived secreted bioactive factors and extracellular vesicles (EVs) [49–51]. Thus, these two major mechanisms including (1) direct MSC differentiation into cardiac and endothelial cells and (2) paracrine activity mediated by MSC-derived soluble molecules and vesicles are currently considered as major factors mediating beneficial effects of MSC-based therapies in CVDs [49–51]. It is believed that most of the beneficial effects after MSC transplantation are related to their paracrine impact on endogenous cells that results in an increase in vasculogenesis and angiogenesis as well as enhanced cell survival.

Although MSCs, as adult cells, represent one of the safest populations of stem cells with nearly no risk of endogenous teratogenic potential characterizing normal pluripotent stem cells such as ESCs and iPS cells, the *in vivo* applications of MSCs into cardiac tissues may still potentially lead to some adverse effects following their transplantation [9, 52, 53]. The few reported concerns regarding MSC safety are related to their possible (1) proarrhythmic and (2) tumorigenic capacity in a heart tissue as well as to (3) differentiation into undesirable tissue type [9, 52, 53]. Price and colleagues reported that BM-MSCs infused intravenously in pigs with acute ischemia/reperfusion injury improves heart function parameters and limits adverse wall thickening, but it may also negatively impact on electrophysiological properties of the myocardium suggesting a proarrhythmic potential of these cells. However, the beneficial effects of injected MSCs on heart function and anatomy observed in this study were greater than the noted arrhythmic events, and eventually the authors positively concluded on the MSC efficacy in their heart repair model [9]. Importantly, on the other hand, multitude clinical studies involving patients suffering with acute or chronic ischemic heart diseases have reported very limited or no adverse effects of MSCs on myocardial electrical properties following the treatment as summarized in several reviews and meta-analysis reports [41, 54]. Thus, the reported events of proarrhythmic activity of MSCs are rather rare; however, it should be considered and evaluated especially in clinical trials with humans as a potential risk indicated by some animal studies. Some of the unsolved problems in clinical research in cardiology may be also partially related to insufficient placebo groups. However, when compared to other stem and progenitor cells used for heart repair including skeletal muscle myoblasts extensively studied in the early 2000s, MSCs may be definitely considered as cells with limited risk of arrhythmia in cardiac tissue [55].

A similar risk of undesirable tissue formation in the sites of MSC transplantation may be related to their innate capacity to predominantly give rise into bone and cartilage cells. Although the unique microenvironment of the cardiac niche consists of several mediators directing stem cell fate and differentiation [56], it has been shown in some single reports that MSCs transplanted into heart tissue may undergo misdifferentiation into other noncardiac cells [52]. Indeed, some experiments conducted on animal models proved episodes of calcification and ossification of heart tissue and injured abdominal aorta after BM-MSC administration [53, 57]. It must be considered that unreliable published data may provide some problems for the field of stem cell practical application as it was in the recent case of cardiac progenitors. Moreover, not carefully designed studies might have a negative influence on the results of clinical trials. Furthermore, several studies underline that effectiveness of therapy may be influenced by quality of stem cells including their senescence status [58].

The other novel approaches to limit the potential risks related to cellular properties of stem cells including MSCs would be a use of their acellular MSC products such as extracellular vesicles (EVs). MSCs are believed to secrete EVs loaded with regulatory factors like miRNA, mRNA, proteins, growth factors, and cytokines. Several recent studies including our own data indicates that MSC-derived EVs represent bioactive specimens released by MSCs that may become a new effective and safe therapeutic tool in heart repair and may gradually replace the whole stem cell-based therapies in the near future [51, 59].

### 3.4. MSCs in Neurology

There is great interest in the establishment of the effective denouement in the field of cell-based therapies with the future application for neurodegenerative diseases. Multiples studies are being undertaken on the administration of MSCs in various neurological diseases so far. However, there is a growing data set that MSC therapy is still ineffective and even might be harmful in some cases. There are already reports from clinical trials stating the absence of the substantial beneficial therapeutic effect of transplanted MSCs.

In the case of amyotrophic lateral sclerosis (ALS), phase I study results did not show notable positive effects of exogenous MSCs deposited to the spinal cord. The main conclusion in most of the clinical trials in ALS therapy with the use of MSCs was limited to the statement concerning safety of a treatment. In a study performed by Syková and colleagues, where BM-MSCs were transplanted intrathecally, a beneficial outcome was visible only in few of patients and was restricted to the short time following transplantation [10]. The cause of such result might be a result of short survival of the cells after implantation or the differentiation state of transplanted MSCs. Repeated transplantation might be crucial in order to achieve better results; however, this fact may be problematic in terms of the manufacturing process. Another phase I study with the intrathecal autologous BM-MSC injection brought light to the mild adverse overcoming reactions immediately after exogenous cell deposition, such as fever, pain, and headache; nevertheless, there were no major effects of therapy [60]. The group of Staff and colleagues performed intrathecal adipose-derived MSC injection in treatment of ALS. Similarly, the authors did not notice any spectacular improvements in treated patients and postulated mitigation in enthusiasm toward the effectiveness of therapy [61]. To add with, intramuscularly and intrathecally transplanted MSCs aspirated from BM were found to be safe and stimulated release of autologous neurotrophic factors; however, such approach contributed to some disease regression only in half of the patients in the following 6-month period [62]. Nevertheless, earlier studies of Karussis and coworkers where BM-derived MSCs were injected intravenously and intrathecally did not bring any positive effects in ALS patients [63].

In case of stroke, one of the main topics related to the efficient cell treatment is the delivery route to the damaged part of the brain. For this reason, the blood circulation system is considered to be the appropriate one. However, one should be aware of the how the administration of the exogenous cells is performed in order to assure the safety for the patient on the one hand and to guarantee the maintenance of the good quality of therapeutic cells during transplantation procedure on the other hand [64]. Cui and colleagues revealed that cell clump formation before injection was increasing proportionally with the increasing time of cell storage duration in suspension. In addition, due to their size, MSCs might elicit serious vascular obstructions after intravascular delivery. The size of MSCs in the monolayer culture *in vitro* increases along with the passage number; a solution may pose 3D spheroid culture *in vitro* that makes MSCs smaller again [65]. The failure of a positive outcome after systemic administration of MSCs in stroke was confirmed by another study, where intravenously injected hBM-MSCs in a mouse stroke model contributed to the enhancement of cell proliferation in neurogenic areas just to a small extent. Furthermore, neither detectable decline in infarct size nor beneficial clinical symptoms were reported [66]. Even more, MSCs intra-arterially delivered in a mouse model of ischemia did not contribute to the functional recovery improvement and additionally might promote the risk of cerebral lesions [67]. These multifocal lesions contributed to the significant cerebral blood flow drop, which was a result of the obstruction of small vessels by the exogenous cells in the circulatory system, conjointly eliciting a profound risk of secondary embolism in stroke brains. These data are reinforced by another study where the intra-arterially delivered human BM-MSCs were transiently present in the injured brain, but this phenomenon was not accompanied with behavioural improvement in a rat stroke model [68]. A similar outcome was observed by Oh and coworkers that intra-arterially delivered MSCs isolated from adipose tissue exerted a just transient effect shortly after their commitment to the target area, and there was no further functional improvement reported; thus, it seemed that cell replacement did not occur [69]. Finally, an intra-arterial route failed to deliver MSCs to the brain parenchyma in an Alzheimer's disease mouse model. Presumably, systematically injected human umbilical cord blood MSCs would not cross the brain blood barrier, which is not injured in this disorder [70].

In the case of multiple sclerosis (MS), an intravenous route of BM-MSC delivery turned out to be not the most satisfactory, because this way of application of the cells failed for mouse MSCs in mouse models of MS. MSCs were found mainly in the lungs and liver with the parallel MSC absence in the inflammatory lesions in the central nervous system. To add with, no beneficial effects were reported [71]. An intra-carotid way of delivery was explored to deliver MSCs to the brain parenchyma in a rat model of Parkinson's disease. However, the number of engrafted BM-derived MSCs in the brain was minimal due to the lack of ability to cross the brain blood barrier by these cells [72]. Even deposited directly to the brain parenchyma, exogenous MSCs isolated from BM miscarried their therapeutic infliction and failed to differentiate into lost neurons and were eliminated up to one month after administration [73]. These results are concordant with the outcome obtained in a mouse model of MS, where human BM-MSCs were implanted intraventricularly or exactly into the lesions of the corpus callosum. MSCs did not modulate positively any regenerative functions and remyelination resulting in no direct beneficial effect [74].

### 3.5. MSCs in Orthopedics

Numerous studies with different animal models of orthopedic diseases have documented the multipotency properties of MSCs, showing their abilities for differentiating into a variety of tissues such as muscle, bone, cartilage, and tendon. However, against the initial assumptions that the therapeutic effects of MSCs depend on their abilities for cell replacement, recent studies showed that paracrine function of MSCs is the main mechanism by which they participate in tissue repair [75]. MSCs have been reported to exhibit immunosuppressive and immunomodulatory properties through the secretion of specific factors that can modulate inflammatory responses after orthopedic injuries [76]. Nevertheless, the widespread mechanism of MSC action in orthopedic applications has not been completely established [77].

Many reports summarized the role of MSCs in osteoarthritis (OA) therapy; however, the results of these studies are quite inconsistent. Recent published data reported that several early-stage clinical trials, testing an intra-articular infusion of MSCs into the knee of patients with OA, were not completed; moreover, the optimal dose and the route of cell administration are still to be established [11]. Filardo and colleagues reported that, despite a growing body of evidence in the biological approach of cartilage regeneration, the understanding of this merit is still insufficient [78]. Further randomized controlled trials are definitely required to estimate the potential of MSCs in cartilage repair and to evaluate advantages and disadvantages of stem cell treatment. Several experiments done in animal models of knee OA have shown that MSC therapy may delay progressive degeneration of the joint [79, 80]. In a rabbit model, it was demonstrated that single intra-articular infusion of synovial MSCs into the knee leads to cellular adherence close to meniscal defect and supported meniscal regeneration [81]. Another study conducted in dogs demonstrated that repeated local delivery of allogeneic MSCs exerted systemic immunomodulatory effects [82]. Most of human studies support the notion that the short-term application of MSCs is safe and feasible; however, further experiments are needed. Importantly, we still need a clear evidence confirming efficacy of MSC transplantation in patients with OA. In randomized controlled clinical trials, MSC injection applied in the treatment of knee osteoarthritis showed its efficacy [83, 84]. Despite these results coming from clinical trials, a recent systematic review by Pas and colleagues does not recommend MSC injections for knee OA because of nonconvincing evidence data. Furthermore, the results quoted by Shim et al. and Pas et al.'s groups revealed that after MSC injections in knee OA treatment, only few cells survived in the place of injection [85, 86]. According to Veronesi et al., the efficacy of MSCs used in the treatment of cartilage disease depends on employed procedure. Moreover, the number of cells may differ between 3.8-11.2 × 10^6^ which determines the time of culture and type of application in osteochondral defects [87]. In addition, an optimal therapeutic dose of cells, coadjuvants, and source of harvesting has not been optimized yet [83]. MSC applications in cartilage repair have an important bias limitation related to the placebo, as the tissue harvesting procedure makes difficult to perform a blinded designed study [78]. Thus, new studies employing MSC-based specimens for orthopedic patients have to be performed with more caution and in controlled ex vivo preparation conditions, to eventually evaluate their therapeutic efficacy in these patients.

Multiple studies suggest that besides the potential effect of MSCs on tissue regeneration, these cells may also be significantly involved in the process of heterotopic ossification (HO), a process of ectopic bone tissue formation in non-bone tissues [88]. Moreover, stem cell therapies in orthopedic trauma have identified MSCs as factors promoting high osteogenic differentiation [89]. Additionally, inflammatory reaction may stimulate the differentiation of mesenchymal progenitor cells (MPCs) into osteoblasts and osteoblast-like cells. If this process is localized in muscles or other soft tissues, it may directly contribute to HO formation [90]. It has been also reported that MSCs could be responsible for the recurrence of HO (after surgical excision). In turn, the excision of HO may result in reemergence of the MSC population and signaling mechanisms that are observed in primary lesion [91].

## 4. Conclusions

Some recent research demonstrated limited therapeutic effects of MSC treatment, suggesting that the direct regenerative potential of these cells related to their differentiation capacity may not be as effective as previously expected. Since several exogenous factors may greatly impact on the MSC biological properties and eventually on their therapeutic abilities, optimized protocols for MSC isolation and ex vivo preparation for clinical use need to be well established and standardized. Such comprehensive effort should be undertaken into consideration by a scientific community focusing on the practical MSC applications in tissue repair in terms of optimal preparation of MSC-based products for more effective therapies in patients.

The advantages of MSC applications in tissue repair, i.e., their safety, relatively wide differentiation capacity, and high paracrine ability including EV release, make these cells an important material for further investigation and development of new approaches for cell-based therapies in future. However, more research studies on both preclinical and clinical levels have to be accomplished. New information related to MSCs will help to determine the efficiency of cells administered to the patients as a therapeutic approach. Additional studies would also be a major contribution to stem cell biology in general.
